# MicroRNA-184 promotes differentiation of the retinal pigment epithelium by targeting the *AKT2*/mTOR signaling pathway

**DOI:** 10.18632/oncotarget.10566

**Published:** 2016-07-13

**Authors:** Chao Jiang, Bing Qin, Guohua Liu, Xiantao Sun, Houxia Shi, Sijia Ding, Yuan Liu, Meidong Zhu, Xue Chen, Chen Zhao

**Affiliations:** ^1^ Department of Ophthalmology, The First Affiliated Hospital of Nanjing Medical University and State Key Laboratory of Reproductive Medicine, Nanjing Medical University, Nanjing, China; ^2^ Department of Ophthalmology, The First People's Hospital of Suqian, Suqian, China; ^3^ Department of Ophthalmology, Qilu Hospital of Shandong University, Jinan, Shandong, China; ^4^ Department of Ophthalmology, Children's Hospital of Zhengzhou, Zhengzhou, China; ^5^ Department of Ophthalmology, Nanjing First Hospital, Nanjing Medical University, Nanjing, China; ^6^ Save Sight Institute, Discipline of Clinical Ophthalmology and Eye Health (CO9), The University of Sydney, Sydney, Australia; ^7^ Department of Ophthalmology, Eye and ENT Hospital, Fudan University, Shanghai, China; ^8^ State Key Laboratory of Ophthalmology, Zhongshan Ophthalmic Center, Sun Yat Sen University, Guangzhou, China

**Keywords:** miR-184, retinal pigment epithelium, differentiation, AKT2, mTOR

## Abstract

Dedifferentiation of retinal pigment epithelium (RPE) cells is a crucial contributing factor to the pathology of retinal degenerative diseases, including age-related macular degeneration (AMD). Herein, we aim to reveal the roles of microRNAs (miRNAs) in RPE dedifferentiation and seek for potential therapeutic targets. Based on the microarray data, miR-184 was sorted out as the most up-regulated signature along with the differentiation from human induced pluripotent stem cells (hiPSC) to RPE cells, suggesting its potential promotive role in RPE differentiation. *In vitro* study indicated that miR-184 insufficiency suppressed RPE differentiation, typified by reduction of RPE markers, and promoted cell proliferation and migration. The role of miR-184 in maintaining regular RPE function was further proved in zebrafish studies. We also noticed that miR-184 expression was reduced in the macular RPE-choroid from a donor with RPE dysfunction compared to a healthy control. We next demonstrated that RAC-beta serine/threonine-protein kinase (*AKT2*) was a direct target for miR-184. MiR-184 promoted RPE differentiation via suppression of AKT2/mammalian target of rapamycin (mTOR) signaling pathway. We also found that *AKT2* was up-regulated in macular RPE-choroid of the donor with RPE dysfunction and dry AMD patients. Taken together, our findings suggest that miR-184 insufficiency is involved in the pathogenesis of dry AMD. MiR-184 promotes RPE differentiation via inhibiting the AKT2/mTOR signaling pathway. MiR-184 based supplementary therapeutics and mTOR blocker, like rapamycin, are prospective options for AMD treatment.

## INTRODUCTION

Retinal pigment epithelium (RPE) is a cuboidal, polarized, and pigmented epithelial cell layer located in the outer retina between the light-sensitive outer segments of photoreceptors and choroidal vasculature forming a part of the blood/retina barrier [1, 2]. As a monolayer of pigmented cells, RPE is crucial in maintaining retinal functions, including absorbing light energy focused by the lens onto the retina; transporting nutrients and ions between photoreceptors and choriocapillaris; regulating ion balance in sub-retinal space; exchanging, storing, and enzymatic conversing retinoid essential for visual cycle; phagocytosis of shed photoreceptor membranes; and secretion of a variety of growth factors [2, 3]. Irregular RPE function may interrupt retinal homeostasis, induce photoreceptor degeneration, generate choroidal neovascularization, and result in vision loss. Dysfunction and depletion of RPE are therefore considered as hallmarks for both monogenic and polygenic retinal degenerative disorders [2, 4–6].

Age-related macular degeneration (AMD), among the most common polygenic retinal degenerative diseases, is a worldwide leading cause for irreversible visual impairments in people aged over 55 [7–9]. AMD can be categorized into two forms, atrophic and exudative [9]. Abnormal RPE behaviors play a preliminary causative role in both forms [10, 11]. Atrophic AMD is characterized by subepithelial deposits and degeneration of RPE and photoreceptors involving but not limited to the macular region [8]. No efficient treatment has been raised for atrophic AMD. Epithelium dedifferentiation, typified by reduction of RPE markers and cellular hypertrophy, has been identified as a crucial process in atrophic AMD [1]. Thus, it is likely that blocking RPE dedifferentiation may permit vision recovery and become potential therapeutic targets for atrophic AMD.

MicroRNAs (miRNAs) are small (19-25 nucleotides) noncoding regulatory RNA molecules that regulate gene expression by binding to particular sites within the 3′-untranslated region (3′-UTR) of their target mRNAs, repressing or seldom activating target gene expression through modulation of mRNA stability or translation [12–14]. Complexity in the regulatory network between miRNAs and their processing factors has, to certain extents, limits comprehensive and thorough investigations. MiRNAs’ roles in the survival and function of RPE cells have been previously annotated, while their roles in RPE differentiation are still unclear [15, 16]. *MiR-184*, located on 15q25.1, is an evolutionarily conserved non-coding RNA oligo. Mature miR-184, containing 22 nucleotides, shows tissue and developmental stage specific expression patterns [17, 18]. It is selectively enriched in mouse brain, mouse corneal epithelium, zebrafish lens and human RPE [19], and is involved in neurological development, apoptosis, and cell differentiation [20, 21]. Previous study has revealed that miR-184 expression is decreased in RPE of AMD donors [19]; however, its role in AMD pathogenesis, especially in RPE dedifferentiation, is still largely unknown. In this study, we aim to analyze the effect of miR-184 in RPE differentiation.

## RESULTS

### Generation of hiPSC-RPE

**Figure 1:**
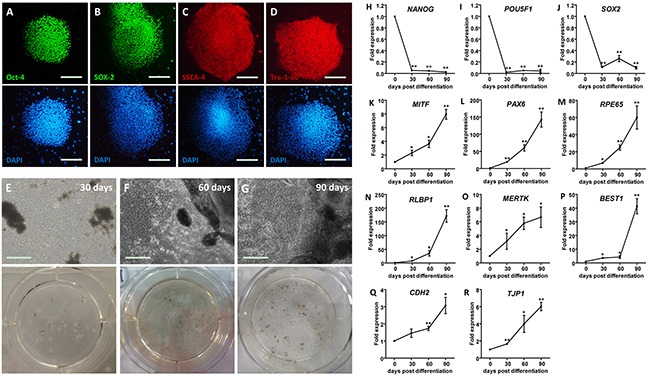
Generation of hiPSC-RPE **A-D.** Immunofluorescent staining of hiPSC. Cell nuclei are presented with DAPI. Images are shown as anti-Oct-4 (A), anti-SOX-2 (B), anti-SSEA-4 (C), and anti-Tra-1-60 (D). **E-G.** Morphological changes of hiPSC-RPE at 30 (E), 60 (F), and 90 (G) days post differentiation (dpd), respectively. **H-R.** mRNA expressions of pluripotent genes, including *NANOG* (H), *POU5F1* (I), and *SOX2* (J), and RPE markers, including *MITF* (K), *PAX6* (L), *RPE65* (M), *RLBP1* (N), *MERTK* (O), *BEST1* (P), *CDH12* (Q), and *TJP1* (R), in hiPSC-RPE at 0, 30, 60, and 90 dpd. *, *p* < 0.05; **, *p* < 0.01.

Pluripotency of the undifferentiated human induced pluripotent stem cells (hiPSC) colonies was confirmed via positive immunostaining for four pluripotency relevant markers, including octamer-binding protein 4 (Oct-4) (Figure 1A), transcription factor SOX-2 (Figure 1B), stage-specific embryonic antigen-4 (SSEA-4) (Figure 1C), and Tra-1-60 (Figure 1D). The hiPSC derived RPE (hiPSC-RPE) was obtained using the SFEB/CS method with small molecule compounds CKI-7 and SB-431542 [22]. Pigmented clusters appeared at 30 days post differentiation (dpd) (Figure 1E), and increased in both volume and quantity along with the differentiation (Figures 1F and 1G).

To determine the competence of differentiation, expression levels of pluripotency-related and RPE signature genes were measured in and compared among undifferentiated hiPSC and hiPSC-RPE at 30, 60, and 90 dpd. Reductions in the mRNA levels of three major pluripotency relevant genes, including homeobox protein NANOG (*NANOG*; NM_024865) (Figure 1H), POU domain class 5 transcription factor 1 (*POU5F1*; NM_002701) (Figure 1I), and *SOX2* (NM_003106) (Figure 1J), were noticed in the differentiated hiPSC-RPE, suggesting its loss of pluripotency. RPE markers were gained through differentiation. Expression levels of the following RPE markers consistently elevated along with the differentiation: RPE-related and developmental transcription factors including microphthalmia-associated transcription factor (*MITF*; NM_198159) (Figure 1K) and paired box protein pax-6 (*PAX6*; NM_001127612) (Figure 1L); visual cycle genes including retinoid isomerohydrolase (*RPE65*; NM_000329) (Figure 1M) and retinaldehyde-binding protein 1 (*RLBP1*; NM_000326) (Figure 1N); membrane proteins and channels including tyrosine-protein kinase Mer (*MERTK*; NM_006343) (Figure 1O) and bestrophin-1 (*BEST1*; NM_001139443) (Figure 1P); and junctional integrity and migration relevant genes cadherin-2 (*CDH2*; NM_001792) (Figure 1S) and tight junction protein ZO-1 (*TJP1*; NM_003257) (Figure 1T).

### Expression profiles of miRNAs regulating the RPE differentiation

**Figure 2:**
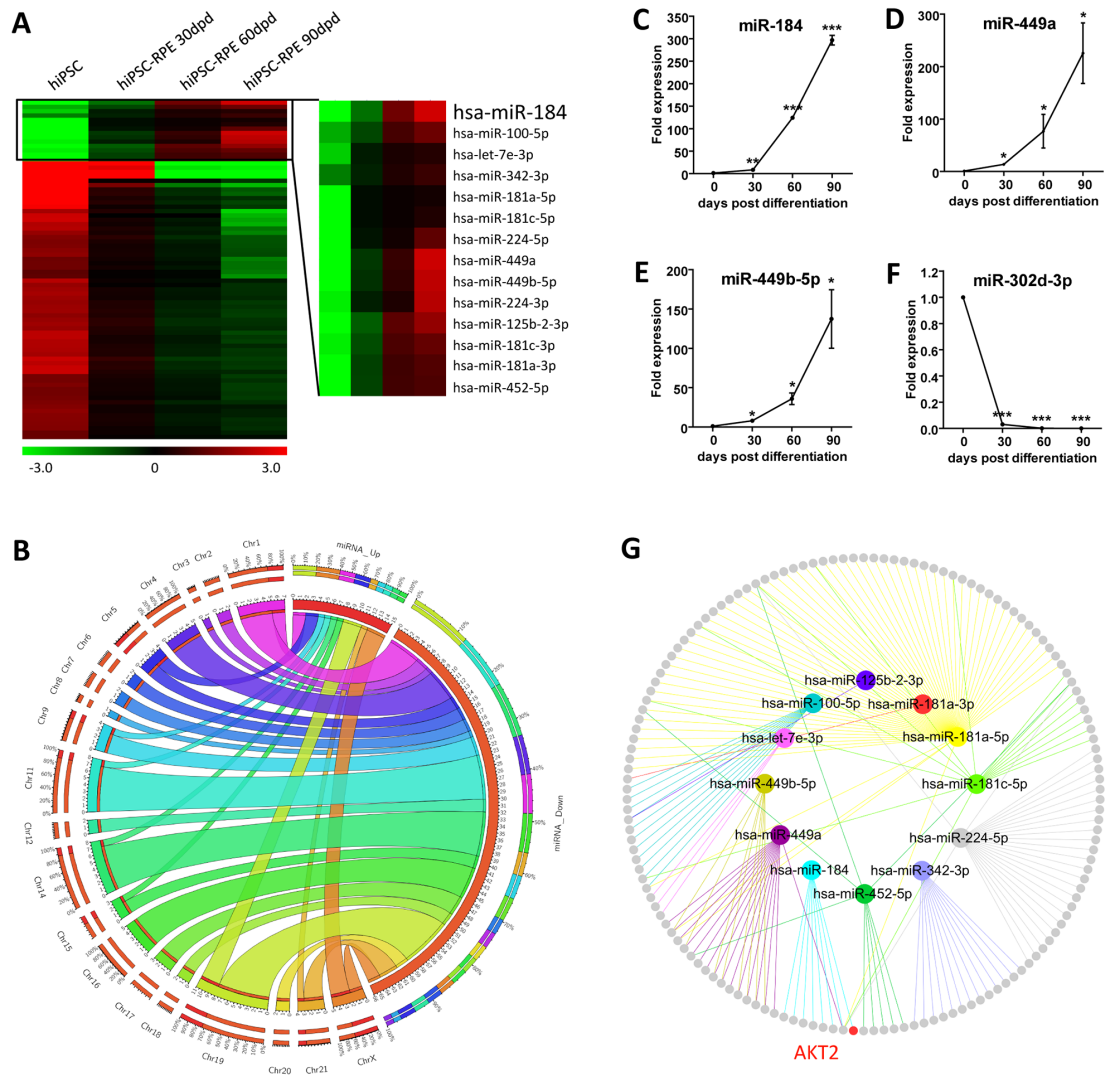
Expression profiling of miRNAs **A.** Hierarchical clustering of all 78 concordantly differentially expressed miRNAs. Hsa-miR-184 was constantly up-regulated along with the differentiation from hiPSC to RPE. Green: down-regulated miRNAs; red: up-regulated miRNAs. **B.** Chromosomal locations of the 78 differentially expressed miRNAs were presented as a Circos plot. **C-F.** Four differentially expressed miRNAs with fold change of over 2 among all time points, including the up-regulated hsa-miR-184 (C), hsa-miR-449a (D), hsa-miR-449b-5p (E), and the down-regulated hsa-miR-302d-3p (F), were selected for validation of the microarray results in hiPSC-RPE at 0, 30, 60, and 90 dpd. (G) Network among all up-regulated miRNAs and their respective experimentally confirmed mRNA targets. The upregulated miRNAs were labeled. Colored nodes: miRNAs; grey nodes: mRNAs; colored edges: miRNA-target interaction.*: p<0.05; **: p<0.01; ***: p<0.001.

To study the role of miRNAs in RPE differentiation, we used microarray analysis to characterize the expression profiles of miRNAs in hiPSC and hiPSC-RPE at 30, 60, and 90 dpd, respectively. All identified miRNAs were initially filtered by fold change. Only miRNA with a fold change of over 2 in the 30 dpd hiPSC-RPE compared to the undifferentiated hiPSC was included. MicroRNA expressions changed consistently at all time points along with the differentiation were considered as crucial for hiPSC differentiation and sorted out. A total of 78 differentially expressed miRNAs, including 14 up-regulated and 64 down-regulated, were selected with their chromosomal locations annotated (Figures 2A-2B and Supplementary Table S1).

To validate the microarray data, we picked four differentially expressed miRNAs with a consistent fold change of over 2 along with the differentiation and analyzed their expressions using real-time polymerase chain reaction (PCR). Agreed with the microarray data, hsa-miR-184, hsa-miR-449a and hsa-miR-449b-5p were consistently up-regulated along with the differentiation, while hsa-miR-302d-3p was down-regulated (Figures 1C-1F). mRNAs targeted by the up-regulated miRNAs with experimental supports were then obtained using miRTarBase (Figure 2G and Supplementary Table S2) [23]. RAC-beta serine/threonine-protein kinase (*AKT2*; NM_001626) was found as a target for hsa-miR-184 (Figure 2G) [24].

### Hsa-miR-184 promotes RPE differentiation

**Figure 3:**
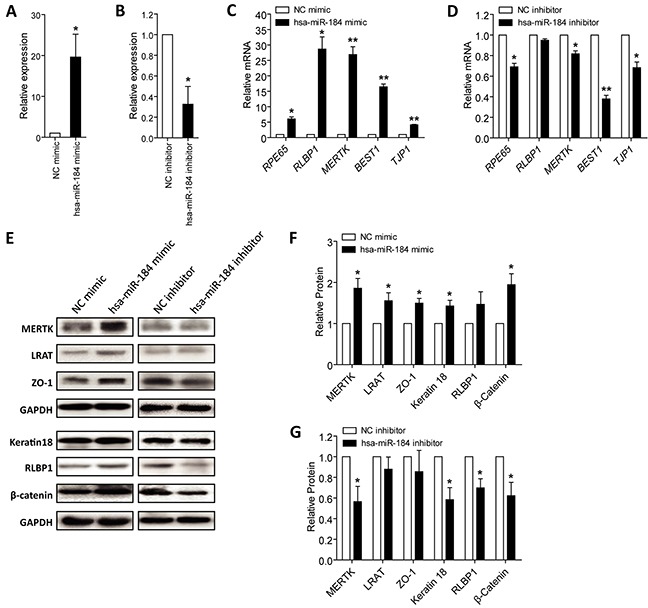
Hsa-miR-184 promotes cell differentiation **A-B.** Relative miRNA expression of hsa-miR-184 in hiPSC-RPE at 30 dpd transfected with hsa-miR-184 mimic compared to NC mimic (A), and in cells transfected with hsa-miR-184 inhibitor compared to NC inhibitor (B). **C-D.** Relative mRNA expressions of *RPE65*, *RLBP1*, *MERTK*, *BEST1*, and *TJP1* in hiPSC-RPE at 30 dpd transfected with hsa-miR-184 mimic compared to NC mimic (C), and in cells transfected with hsa-miR-184 inhibitor compared to NC inhibitor (D). **E-G.** Relative protein expressions of MERTK, LRAT, ZO-1, Keratin 18, RLBP1, and β-catenin in hiPSC-RPE at 30 dpd transfected with hsa-miR-184 mimic compared to NC mimic (E-F), and in cells transfected with hsa-miR-184 inhibitor compared to NC inhibitor (E, G). *: p<0.05; **: p<0.01.

As above mentioned, hsa-miR-184 expression was consistently increased along with RPE differentiation (Figure 1C), we then respectively transfected hsa-miR-184 mimic and inhibitor into hiPSC-RPE at 30 dpd to see its role on cell differentiation. Hsa-miR-184 mimic comprises chemically synthesized oligonucleotides identical to the sequence of endogenous hsa-miR-184, which will be loaded into the RNA-induced silencing complex (RISC) and silence target genes like endogenous hsa-miR-184 [25]. Antisense hsa-miR-184 oligonucleotides are used as hsa-miR-184 inhibitor, which binds directly to the single strand mature hsa-miR-184 to block its activity [26]. Exogenous hsa-miR-184 expression was remarkably up-regulated in cells transfected with hsa-miR-184 mimic (Figure 3A), and endogenous hsa-miR-184 expression was down-regulated in cells transfected with hsa-miR-184 inhibitor (Figure 3B). Expressions of several RPE markers were then tested, including mRNA expressions of *RPE65*, *RLBP1*, *MERTK*, *BEST1* and *TJP1*, and protein expressions of MERTK (encoded by *MERTK*; NP_006334), lecithin retinol acyltransferase (encoded by *LRAT*, NM_004744; NP_004735), ZO-1 (encoded by *TJP1*; NP_003248), keratin type I cytoskeletal 18 (encoded by *KRT18*, NM_000224; NP_000215), RLBP1 (encoded by *RLBP1*; NP_000317), and β-catenin (encoded by *CTNNB1*; NP_001895). Ectopic hsa-miR-184 overexpression elevated both mRNA and protein expressions of the RPE markers (Figures 3C, 3E-3F), while hsa-miR-184 insufficiency suppressed their expressions (Figures 3D, 3E, and 3G). Our findings suggested that hsa-miR-184 promotes the differentiation of hiPSC-RPE.

### Dre-miR-184 insufficiency suppresses RPE development *in vivo*

**Figure 4:**
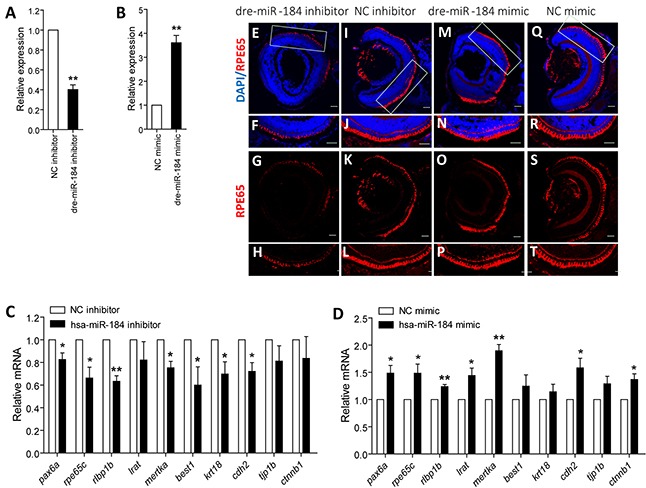
Dre-miR-184 promotes retinal development *in vivo* **A-B.** Relative expression of dre-miR-184 in zebrafish injected with dre-miR-184 inhibitor compared to NC inhibitor (A), and in embryos injected with dre-miR-184 mimic compared with NC mimic (B). **C-D.** Relative mRNA expressions of *pax6a*, *rpe65c*, *rlbp1b*, *lrat*, *mertka*, *best1*, *krt18*, *cdh2*, *tjp1b*, and *ctnnb1* in zebrafish injected with dre-miR-184 inhibitor compared to NC inhibitor (C), and in embryos injected with dre-miR-184 mimic compared with NC mimic (D). *: p<0.05; **: p<0.01.

We next used zebrafish model to investigate the role of dre-miR-184 on RPE development. Embryos at 1- to 2-cell stage were individually injected with dre-miR-184 mimic or inhibitor to modulate its exogenous or endogenous dre-miR-184 expression (Figures 4A-4B). Embryos injected with negative control (NC) inhibitor or mimic were taken as controls. mRNA expression of RPE markers, including *pax6a* (NM_131304), *rpe65c* (NM_001113653), *rlbp1b* (NM_205690), *lrat* (NM_001135971), *mertka* (XM_002664231.4), *best1* (XM_009297704.1), *krt18* (NM_178437), *cdh2* (NM_131081), *tjp1b* (XM_009298138.1), and *ctnnb1* (NM_131059), were tested and compared in zebrafish from all injection groups with normal systemic appearance at 4 day post fertilization (dpf). Q-PCR results indicated that knocking down of dre-miR-184 will suppress the expression of RPE markers, while overexpression of dre-miR-184 can promote RPE development (Figures 4C-4D). Consistent to mRNA findings, immunofluorescence revealed decreased reactivity of retinoid isomerohydrolase (encoded by *rpe65c*; NP_000320), a marker of RPE cells, in the RPE layer of zebrafish injected with dre-miR-184 inhibitor (Figures 4E-4H) compared to embryos injected with NC inhibitor (Figures 4I-4L). By contrast, the dre-miR-184 mimic injection group showed robust expression of retinoid isomerohydrolase in the RPE layer (Figures 4M-4P), similar to the NC mimic injection group (Figures 4Q-4T). Our findings suggested that dre-miR-184 functions in maintaining the regular function of zebrafish RPE, and its insufficiency would suppress RPE development.

### *AKT2* is a direct target of hsa-miR-184 in RPE cells

**Figure 5:**
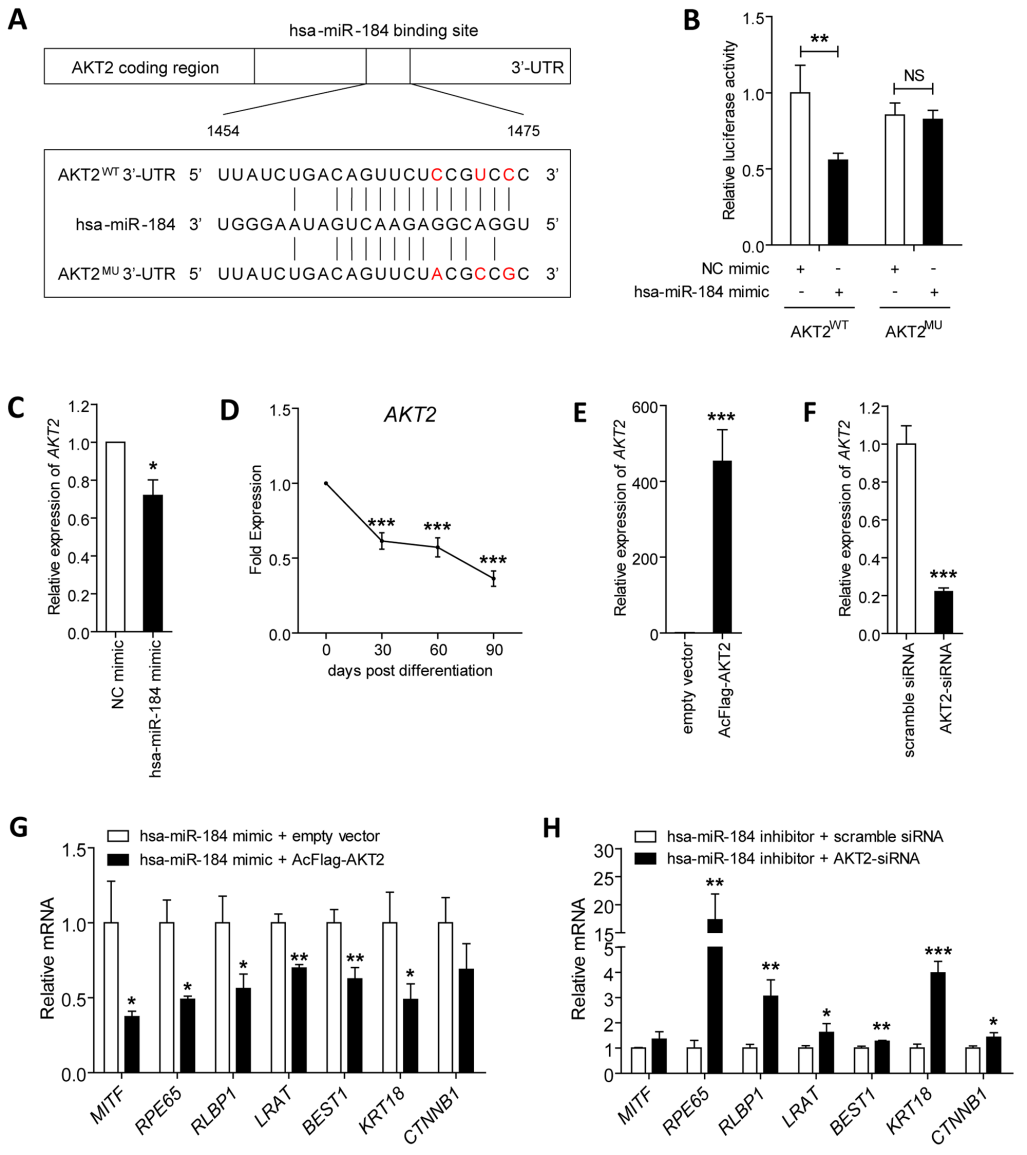
Hsa-miR-184 targets the *AKT2*/mTOR pathway, and inhibits cell proliferation and migration **A.** Schematic construction of reporter containing a fragment of the wild type and mutant *AKT2* 3′-TUR. **B.** Relative luciferase activities were measured in ARPE-19 cells. Renilla luciferase vector was used as an internal control. **C.** mRNA expression of *AKT2* were analyzed in 30 dpd hiPSC-RPE transfected with NC mimic or hsa-miR-184 mimic, respectively. **D.** *AKT2* levels were determined in hiPSC-RPE at 30, 60, and 90 dpd. **E-F.** *AKT2* expression was elevated in ARPE-19 cells transfected with AcFlag-AKT2 (E), and was suppressed in cells transfected with AKT2-siRNA (F). **G-H.** Relative mRNA expressions of *MITF*, *RPE65*, *RLBP1*, *LRAT*, *BEST1*, KRT18, and *CTNNB1* in hiPSC-RPE at 30 dpd transfected with hsa-miR-184 mimic plus empty expression vector compared to hsa-miR-184 mimic plus AcFlag-AKT2 (G), and in cells transfected with hsa-miR-184 inhibitor plus scramble siRNA compared hsa-miR-184 inhibitor plus AKT2-siRNA (H). *: p<0.05; **: p<0.01; ***: p<0.001.

**Figure 6:**
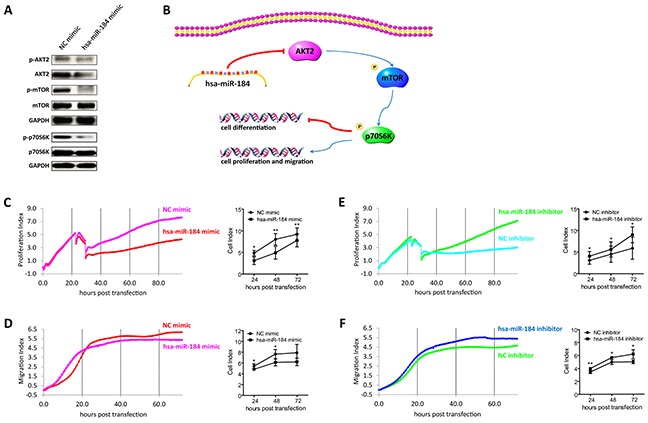
Hsa-miR-184 suppresses the AKT2/mTOR pathway, and inhibits cell proliferation and migration **A.** Immunoblotting of p-AKT2, AKT2, p-mTOR, mTOR, p-p70S6K, p70S6K in 30 dpd hiPSC-RPE transfected with NC mimic/hsa-miR-184 mimic. **B.** Schematic of the hsa-miR-184 regulatory network. **C-F.** Proliferative rates of ARPE-19 cells transfected with NC mimic (C), hsa-miR-184 mimic (C), NC inhibitor (E), and hsa-miR-184 inhibitor (E), and migration of ARPE-19 cells transfected with NC mimic (D), hsa-miR-184 mimic (D), NC inhibitor (F), and hsa-miR-184 inhibitor (F).

Previous study suggested that hsa-miR-184 suppressed *AKT2* expression in neuroblastoma [24]. We then focused on studying whether *AKT2* is a potential target of hsa-miR-184 in RPE cells. Initial assessments were carried out to see whether *AKT2* expression levels are inversely related to hsa-miR-184 levels. *AKT2* expression was found consistently decreased along with the differentiation from hiPSC to RPE as demonstrated in Figure 5D, which was inversely correlated to the expression pattern of hsa-miR-184 (Figure 2C). To confirm that *AKT2* was inhibited by hsa-miR-184 in RPE, we next measured the expression of *AKT2* in 30 dpd hiPSC-RPE transfected with hsa-miR-184 mimic. Q-PCR analysis revealed that overexpression of exogenous hsa-miR-184 down-regulates the *AKT2* mRNA level in 30 dpd hiPSC-RPE (Figure 5C). The same result was observed at protein level (Figure 6A).

We further aimed to tell whether the effect of hsa-miR-184 on *AKT2* expression is a direct effect of hsa-miR-184 targeting *AKT2* 3′-UTR using luciferase reporter assay. A fragment from the 3′-UTR of the *AKT2* gene bearing its binding region with hsa-miR-184 was cloned into the firefly pMIR-GLO™ luciferase vector (pMIR, Invitrogen) to construct the recombinant plasmid AKT2^WT^ (Figure 5A). Luciferase activity was significantly decreased in ARPE-19 cells co-transfected with the AKT2^WT^ plasmid and hsa-miR-184 mimic compared to cells co-transfected with the reporter and NC mimic (Figure 5B). However, introduction of 3 single nucleotides located in the core binding region of *AKT2* completely abolished the ability of hsa-miR-184 mimic to decrease luciferase activity (Figure 5B). Altogether, our findings supported that hsa-miR-184 directly targets *AKT2* 3′-UTR and suppresses *AKT2* expression in RPE.

### Hsa-miR-184 promotes RPE differentiation via suppression of *AKT2*

We next tested whether *AKT2* could affect the promotive effect of hsa-miR-184 on RPE differentiation. The efficiency of AcFlag-AKT2 in overexpressing *AKT2* (Figure 5E) and AKT2-siRNA in silencing *AKT2* were initially confirmed in ARPE-19 cells (Figure 5F). Expression of several mRNAs were then tested and compared, including *MITF*, *RPE65*, *RLBP1*, *LRAT*, *BEST1*, *KRT18*, and *CTNNB1*. We showed that *AKT2* overexpression abrogated the hsa-miR-184 mediated RPE differentiation (Figure 5F). Further, silencing of *AKT2* rescued the inhibition on RPE differentiation induced by hsa-miR-184 insufficiency (Figure 5F). Collectively, our data implied that hsa-miR-184 promotes RPE differentiation via suppression of *AKT2*.

### Hsa-miR-184 suppresses the AKT2/mTOR pathway, and inhibits cell proliferation and migration

We have previously reported that RPE dedifferentiation could arise through stimulation of the AKT/mammalian target of rapamycin (mTOR) pathway [1]. As indicated above, hsa-miR-184 would promote RPE differentiation, and *AKT2* was a direct target of hsa-miR-184. We therefore hypothesized that hsa-miR-184 might promote RPE differentiation via blocking the AKT2/mTOR signaling pathway (Figure 6B). As shown in Figure 6A, western blot analysis revealed decreased phosphorylation of AKT2^Ser474^, mTOR^Ser2448^, and p70S6K^Thr389^ in 30 dpd hiPSC-RPE overexpressing hsa-miR-184, suggesting that hsa-miR-184 could inhibit the AKT2/mTOR pathway. Since cellular proliferation and migration can follow the dedifferentiation of postmitotic tissues, including RPE, and activation of mTOR pathway have been reported to enhance cell proliferation and migration [16, 27–30], we next determined whether hsa-miR-184 would inhibit proliferation and migration of ARPE-19 cells. Cell proliferative and migratory rates were continuously detected till 72 hours (hr) post transfection. Both proliferation and migration were inhibited by hsa-miR-184 overexpression at all time points post transfection (Figures 6C-6D), and cell proliferative and migratory rates were increased in cells with endogenous hsa-miR-184 down-regulated (Figures 6E-6F). Taken together, our results suggested that hsa-miR-184 suppresses the AKT2/mTOR signaling pathway, and inhibits cell proliferation and migration.

### Hsa-miR-184 is decreased in dysfunctional RPE, and *AKT2* is increased in AMD

**Figure 7:**
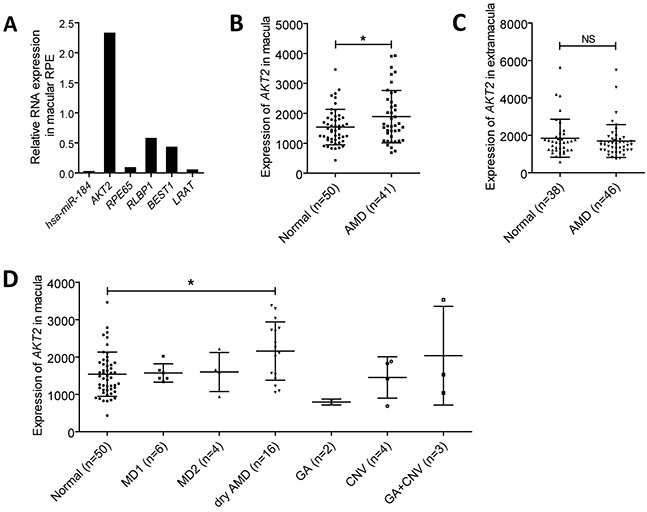
Hsa-miR-184 is down-regulated in dysfunctional RPE, and *AKT2* is up-regulated in AMD **A.** Relative RNA expressions of hsa-miR-184, *AKT2*, *RPE65*, *RLBP1*, *BEST1*, and *LRAT* in the macular RPE of an aged donor compared to a young donor. **B-C.** Expressions of *AKT2* were measured and compared in the macular (B) and extramacular (C) RPE-choroid of age related macular degeneration (AMD) patients and the control group. **D.** *AKT2* expressions were further analyzed and compared in the RPE-choroid of patients with different AMD subtypes.

Activation of mTOR signaling pathway is reported to cause RPE dedifferentiation and retinal degenerative diseases, namely AMD [1]. Since hsa-miR-184 could inhibit mTOR, we, therefore, assumed that hsa-miR-184 expression will be reduced in dysfunctional RPE. To test our hypothesis, we compared the expression of hsa-miR-184 between the macular RPE of a 70-year-old male and a 30-year-old female donor. Although the male donor hadn't been clinically diagnosed with AMD before he passed away, mRNA expressions of RPE markers, including *RPE65*, *RLBP1*, *BEST1* and *LRAT*, were found significantly decreased in his RPE compared to the younger donor, suggesting dysfunction of RPE cells (Figure 7A). As expected, hsa-miR-184 level was found remarkably down-regulated in the aged donor with RPE dysfunction (Figure 7A). Further analysis suggested that, opposite to the decreased hsa-miR-184 level, *AKT2* mRNA was elevated in the macular RPE of the above mentioned aged donor with RPE dysfunction (Figure 7B). Our results were consistent with the previous report that hsa-miR-184 expression was inhibited in the RPE of donors with AMD [19], and further implied that hsa-miR-184 may play a role in keeping regular function of RPE.

Since hsa-miR-184 was reported to present a decreased expression pattern in AMD patients, we next tested and compared the macular and extramacular *AKT2* mRNA levels in RPE-choroid from normal individuals and AMD patients. Summary mRNA data (GSE29801) and sample information (GSM738433 to GSM738607) of 175 independent RPE-choroid samples were downloaded from Gene Expression Omnibus (GEO) datasets and double-checked. The 175 samples contained 50 macular and 38 extramacular RPE-choroid tissues from normal individuals, and 41 macular and 46 extramacular RPE-choroid samples from AMD patients. *AKT2* was found elevated in the macular RPE-choroid of AMD patients compared to healthy controls (Figure 7B), while no expressional difference between the two groups was detected in the extramacular RPE-choroid (Figure 7C). Subgroup analysis was next performed to better tell with which AMD subtype *AKT2* is most closely correlated. Clinical diagnostic criterion for each AMD subgroup has been detailed before [31]. According to our findings, *AKT2* was only found elevated in the dry AMD group (n=16) (Figure 7D), a branch of atrophic AMD. Our results implied a potential role of *AKT2* in atrophic AMD.

## DISCUSSION

RPE dedifferentiation, typified and accompanied with morphological and molecular changes of the RPE cells, has been proved as an important contributing factor to the pathophysiology of retinal degenerative disease, like AMD [1]. Thus, inhibition of RPE dedifferentiation could be a potential therapeutic target for such diseases. MicroRNAs are important regulators in gene expression, and their roles in keeping the survival and regular functions of RPE cells have been well established: conditional deletion of the miRNA processing factor Dicer1 in RPE generates cell death [32], and deficient miR-204/211 levels causes RPE dysfunction and demorphogenesis [16]. Furthermore, miRNAs-based therapeutic strategies, like miRNA replacement therapies, have inherent advantages over other therapeutics in drug efficiency and delivery [33]. In this study, we generate the hiPSC-RPE model to assess the involvement of miRNAs in modulating RPE differentiation. A total of 78 miRNAs are initially sorted out, among which miR-184 is the most up-regulated signature along with the differentiation, and its crucial role in RPE differentiation is further confirmed in both cellular and zebrafish models. We have also reasoned that miR-184 suppresses the AKT2/mTOR signaling pathway, promotes RPE differentiation, and inhibits RPE proliferation and migration.

The role of miR-184 in AMD pathogenesis, especially in RPE dedifferentiation, is still largely unknown. Herein, consistent to previous findings, we show that miR-184 expression is remarkably reduced in the macular RPE of a donor with RPE dysfunction, indicating its role in maintaining regular functions of RPE cells. The impact of miR-184 on promoting RPE development is further confirmed in zebrafish model. Reduction in RPE markers is observed in embryos with miR-184 silenced. Cellular model also proves that the expression level of miR-184 associates with RPE differentiation. Overexpression of miR-184 promotes RPE differentiation, while miR-184 insufficiency could suppress RPE differentiation.

Ezrin (*EZR*) gene is the only reported target of miR-184 involved in RPE functions. Binding of ezrin to lysosomal-associated membrane protein 1 (LAMP-1) is an important process to form phagocytic vacuoles. Reduction in miR-184 expression will decrease level of EZR-bounded LAMP-1 and interrupt RPE phagocytosis [19]. *AKT2* is homolog 2 of the v-akt oncogene, a major downstream effector of the phosphatidylinositol 3′ kinase (PI3K) pathway that can activate the mTOR pathway [34]. Activation of the AKT/mTOR pathway can stimulate RPE dedifferentiation, proliferation, migration and hypertrophy, and is thus supposed to be a crucial disease process for AMD [1]. *AKT2* has been proved as a target for miR-184 in neuroblastoma cell line [24], while their interaction in RPE cells remains inclusive. In the present study, we confirm that *AKT2* is a direct target for miR-184 in ARPE-19 cells, and miR-184 promotes RPE differentiation via suppression of *AKT2*. *AKT2* is found down-regulated in the macular RPE-choroid of AMD patients, especially in patients with dry AMD. We also demonstrate that miR-184 blocks the AKT2/mTOR signaling pathway and suppresses cell proliferation and migration.

Other than retinopathy, miR-184 seed region mutations are reported to cause EDICT syndrome, presenting familial keratoconus with cataract [35, 36]. The mutated miR-184 antagonizes miR-205 to maintain the expression of the inositol polyphosphate phosphatase-like 1 (*INPPL1*) gene, which generates the disease phenotype [19]. This finding implies that in order to sustain RPE function and survival, miR-184 may also modulate the expressions of regulatory factors, like other miRNAs and long non-coding RNAs. In addition, abnormal methylation status of *MIR-184* has also been found correlated with Rett syndrome, presented with autism spectrum disorders and involving irregular synaptic plasticity [18]. Thus, deep investigations into the regulatory network and epigenetic regulation of miR-184 in maintaining RPE function may become part of our future work.

Taken together, our study concludes that miR-184 promotes RPE differentiation via inhibiting the AKT2/mTOR signaling pathway, and miR-184 insufficiency plays an important role in the pathogenesis of dry AMD. MiR-184-based supplementary therapeutics and mTOR pathway inhibitor, like rapamycin, can inhibit RPE dedifferentiation in dry AMD and may become prospective options for treating retinal degenerative diseases. Further work is still warranted to better elucidate the role of miR-184 in AMD pathogenesis.

## MATERIALS AND METHODS

### Samples

Post-mortem specimens of a 70-year-old male donor and a 30-year-old female donor were provided by Lions New South Wales Eye Bank through Save Sight Institute, the University of Sydney, Australia. Written informed consents were obtained from all donors before donation. All procedures followed standard procedures of eye donation for research and were approved by the institutional ethical committees conformed to Declaration of Helsinki.

### Animals

Tuebingen zebrafish were housed in the Model Animal Research Center (MARC), Nanjing University, in accordance with the IACUC-approved protocol. Rearing and husbandry of zebrafish were maintained at 28.5°C in a 14 hr light/10 hr dark cycle. Embryos were produced by natural mating. Animal experiments were approved by the local ethical committees. All procedures were conformed to the Guide for the Care and Use of laboratory animals.

### Culture and differentiation of hiPSC

The hiPSC (IMR90-57, a kind gift from Prof Guoping Fan, Tongji University, Shanghai, China) were cultured on mouse embryonic fibroblasts (MEFs; SiDan-Sai Biotechnology Co., Ltd, Shanghai, China) in six-well tissue culture plates (Corning Glass Works, Corning, New York, USA), and were maintained in Dulbecco's modified Eagle's medium/Ham's F12 nutrient medium 1:1 (DME/F12 medium; Invitrogen, Carlsbad, CA, USA) supplemented with knockout serum replacement (20%; Gibco, Carlsbad, CA, USA), β-mercaptoethanol (0.1 mM; Sigma-Aldrich; St. Louis, MO, USA), L-glutamine (2mM; Gibco), non-essential amino acids (0.1 mM; Gibco), and zebrafish basic fibroblast growth factor (zbFGF; 100 ng/ml; R&D Systems Inc., Minneapolis, Minnesota, USA) at 37°C, 5% CO_2_. *In vitro* differentiation of RPE cells from hiPSC was performed using a previously described SFEB/CS method [22]. We added low-molecular-weight compounds CKI-7 (5 μM) and SB-431542 (5 μM) during suspension culture to block Wnt and Nodal signaling, respectively. These two small molecules are perfect substitutions for recombinant Dkk1 and Lefty-A proteins which were confirmed to induce the differentiation of ES cells into retinal progenitors [37].

### RNA extraction, RT-PCR, and real-time PCR

RNA isolation, cDNA synthesis, and real-time PCR were performed as previously described [3, 38]. Briefly, total RNA was extracted using TRIzol reagent (Invitrogen) according to the manufacturer's instructions. Concentration and quality of RNA samples were measured with Nano-Drop ND-1000 spectrophotometer (Nano-Drop Technologies, Wilmington, DE). One μg of total RNA was used for reverse transcription PCR (RT-PCR) using PrimeScript RT Kit (Takara, Otsu, Shiga, Japan). Real-time PCR was conducted using FastStart Universal SYBR Green Master (ROX; Roche, Basel, Switzerland) with the StepOne Plus Real-time PCR System (Applied Biosystems, Darmstadt, Germany). Primer information was detailed in Supplementary Table S3.

### Immunofluorescence

Cells were harvested, fixed with paraformaldehyde (4%) for 30 minutes (min), permeabilized with Triton X-100 (0.5%) for 20 min, blocked in bovine serum albumin (BSA; 1%) for 2 hours (hr), and incubated with designated primary antibodies at 4°C overnight. Details for antibodies were provided in Supplementary Table S4. Cells were then washed with phosphate buffer saline (PBS; 1X) for 15 min and incubated with corresponding fluorescence-conjugated secondary antibodies (1:1000 diluted in 1X PBS; Invitrogen) for 1 hr at room temperature. Cytoskeleton was counterstained by phalloidin (1:40; Invitrogen) for 30 min, and cell nuclei by 4′, 6-diamidino-2-phenylindole (DAPI; Sigma, USA) for 5 min. Images were collected with an inverted fluorescence microscope (Olympus, Tokyo, Japan).

### miRNA and mRNA expression profiling, and computational analysis

GeneChip® miRNA 3.0 Array (Affymetrix Inc, Santa Clara, CA, USA) covering 100% miRBase v17 (www.mirbase.org) in a one-color way was employed to generate the miRNA expression profiles from the following four groups in duet: hiPSC, hiPSC-RPE at 30, 60, and 90 days [39]. RNA molecules were initially tailed and labeled with a labeling kit as described previously [40]. An Affmerix GCS3000 Gene Array Scanner with a high resolution 6g patch was then applied to scan the fluorescence on the array [39]. Thresholding and signaling scaling were generated using appropriate algorithms as detailed before [41]. Statistical analysis of miRNA arrays were carried out using hypothetical testing with one-way analysis of variance (ANOVA) or Student's t-test [41]. mRNAs targeted by miRNAs with strong validation evidence support, including reporter assay, western blot, and qPCR, were obtained from miTarBase (http://mirtarbase.mbc.nctu.edu.tw/).

Summary mRNA data and sample information of 175 independent RPE-choroid samples were downloaded from GEO datasets. The mRNA expression levels were determined with the Agilent Whole Human Genome Microarray *in situ* oligonucleotide array platform (G4112F, Agilent Technologies Inc., Santa Clara, CA, USA). Microarray hybridization, quantification, and normalization were performed as described previously [31]. Data were analyzed and categorized according to different AMD subtypes.

### Reagents, cell transfection, and luciferase reporter assay

Human and zebrafish miRNA mimics and inhibitors, including NC mimic, NC inhibitor, hsa-miR-184 mimic, hsa-miR-184 inhibitor, dre-miR-184 mimic, and dre-miR-184 inhibitor, were purchased from GenePharma Co., Ltd (Shanghai, China). Scramble siRNA (NControl_05815) and AKT2-siRNA were purchased from Ribobio (Guangzhou, China). Sequences of mimics, inhibitors, and siRNAs were provided in Supplementary Table S5. A fragment from the 3′-UTR of the *AKT2* gene bearing its binding region with has-miR-184 were synthesized and cloned into the firefly pMIR-GLO™ luciferase vector (pMIR, Invitrogen) using *SacI* and *XhoI* restriction sites to construct the recombinant plasmid AKT2^WT^ and AKT2^MU^. The AKT2^MU^ construct contained 3 mutated nucleotides located in the core binding region of *AKT2* as presented in Figure 5A. Primers for plasmid construction were designed to match the wild type (WT) or mutant *AKT2* sequence at their 3′ moiety and carry restriction sites for endonucleases at their 5′ end (Supplementary Table S3). The open reading frame (ORF) sequence of *AKT2* was synthesized, amplified, and inserted into the expression vector pCMV-C-Flag (Beyotime, Shanghai, China) using *BamHI* and *XbaI* restriction sites to generate the AcFlag-AKT2 plasmid. Primer information was provided in Supplementary Table S3. Sequences of the constructed plasmids were confirmed by Sanger sequencing.

For transfection assay, cells were seeded into 6-well templates and transfected with 100 pmol mimic/inhibitor/siRNA, and/or 4 μg expression vector, using Lipofectamine^TM^ 2000 Transfection reagent (Invitrogen, Carlsbad, CA, USA) per the manufacturers’ protocol. Cells were harvested at 48 hr post transfection for RNA isolation, and at 72 hr post transfection for protein extraction.

Luciferase reporter assay was conducted in previously described way [38]. For luciferase reporter assay, cells were seeded into 24-well templates and transfected with 16 ng cytomegalovirus (CMV)-Renilla (Promega, Madison, WI, USA), 20 pmol miR-184 mimic or NC-mimic, and 800 ng AKT2^WT^ or AKT2^MU^. Cells were collected 72 hr after transfection for luciferase activities measuring with the dual luciferase system (Promega, Madison, WI, USA) and a GloMax-96 luminometer. Renilla luciferase activities were taken as internal standard indicators for transfection efficiency. Firefly luciferase activities were then normalized to Renilla luciferase activities.

### Immunoblotting

Cells were harvested 72 hr post transfection in ice-cold RIPA buffer (Beyotime, Shanghai, China) containing protease inhibitors cocktail (Roche) for protein extraction. The extracted proteins were then separated by sodium dodecyl sulfate-polyacrylamide gel electrophoresis (SDS-PAGE; 10%) and transferred to a polyvinylidene fluoride membrane (PVDF; Millipore, Billerica, MA, USA). Membranes were blocked for 2 hr at 37°C with 5% skim milk in Tris-buffered saline containing 0.05% Tween 20 (TBST), incubated with designate primary antibodies at 4°C overnight (Supplementary Table S2), washed with tris buffered saline with Tween (TBST) for 15 min, and probed with corresponding horse reddish peroxidase (HRP)-conjugated secondary antibodies (1:10000 diluted in 1X PBS; ICL Inc., Newberg, OR) for 1 hr at room temperature. After 5 times wash with 1X PBS, the blots were then developed by autoradiography with the ECL-Western blotting system (BioRad, Hercules, CA, USA) according to the manufacturers’ protocols. Protein expressions were determined and quantified using Image J software (http://rsb.info.nih.gov/ij/index.html).

### Zebrafish manipulation

Zebrafish manipulation was performed as described previously [38, 42, 43]. Embryos at 1- to 2-cell stage (0 dpf) were grouped and injected with 1 nl solution containing 4 μM the abovementioned NC mimic, NC inhibitor, dre-miR-184 mimic, or dre-miR-184-inhibitor, respectively. Embryos died within 24 hours post injection were excluded. At 4 dpf, only zebrafish with relative normal morphology were included for further investigations. Ninety zebrafish from each injected group was collected and pooled for expressional analyses. RNA isolation, RT-PCR, and real-time PCR were carried out to determine the expression levels of dre-miR-184 and several RPE markers. Primer information was provided in Supplementary Table S1. Immunofluorescent staining was further employed to determine the expression pattern of the RPE specific protein retinoid isomerohydrolase, which was encoded by the *rpe65* gene. Information of antibody was detailed in Supplementary Table S2. Thirty zebrafish with normal systemic appearance were randomly selected from each injected group for immunofluorescent staining assay. Fish was fixed in 4% paraformaldehyde (PFA) overnight at 4°C and dehydrated in 30% sucrose. We then embedded the embryos with optimal cutting temperature solution, frozen in liquid nitrogen for one min, and sectioned using Leica CM1900 cryostat (Leica, Germany). Slides were then used for immunofluorescent staining as mentioned above.

### Monitoring cell proliferation and migration

Rates of cell proliferation and migration were monitored in real-time with the xCELLigence system E-Plate (Roche) per the manufacturer's protocol. For proliferation assay, 5000 ARPE-19 cells were seeded into each well and transfected with corresponding mimic/inhibitor at 24 hr post plantation, whereas, for migration analyses, 40000 cells were planted into each well of the CIM-Plate right after transfection. All cells were cultured with fresh DME/F12 medium. Impedance values for all wells were automatically monitored with the xCELLigence system for duration of 48 hr post transfection and expressed as a CI value. Cell proliferative rates were determined by calculating the slope of the line between two given time points.

### Statistics

GraphPad Prism (version 4.0; GraphPad Software, San Diego, CA, USA) was applied for statistical analysis. All presented data were based on biological triplicates. Student's T-test was used for comparison between different groups. Data were shown as mean ± standard deviation (SD), and P value < 0.05 was considered as statistically significant.

## SUPPLEMENTARY TABLES






