# Supplementary material for: MicroRNA-184 promotes differentiation of the retinal pigment epithelium by targeting the AKT2/mTOR signaling pathway
Source: Oncotarget. 2016 Jul 13;7(32):52340–53. doi: 10.18632/oncotarget.10566 (PMC5239556; doi:10.18632/oncotarget.10566)
Supplement: Supplementary file 1 [file oncotarget-07-52340-s001.pdf]

MicroRNA-184 promotes differentiation of the retinal pigment epithelium by targeting the *AKT2*/mTOR signaling pathway

SUPPLEMENTARY TABLES

Supplementary Table S1: A collection of differentially expressed miRNAs.

See Supplementary File 1

Supplementary Table S2: Validated miRNAs' targets with experimental support

miRNA	mRNAs targeted by miRNAs
hsa-miR-184	*AKT2, BCL2, EIF2C2, EZR, INPPL1, MYC, NFATC2, SOX7*
hsa-miR-449a	*BCL2, CCND1, CCNE2, CDC25A, CDK4, CDK6, E2F3, GMNN, HDAC1, HDAC8, HDAC8, LEF1, MET, NOTCH1, SIRT1, WISP2*
hsa-miR-449b-5p	*CCNE2, CDC25A, CDK4, CDK6, GMNN, HDAC1, MET, SIRT1*
hsa-let-7e-3p	*COPS6, COPS8, GPS1*
hsa-miR-100-5p	*ATM, BMPR2, CTDSPL, EGR2, FGFR3, FLT1, ID1, IGF1R, MMP13, MTOR, PLK1*
hsa-miR-125b-2-3p	*IGF1R*
hsa-miR-181a-3p	*NANOG, ATM, BCL2, BCL2L11, CDKN1B, CDX2, DDIT4, DUSP5, DUSP6, FOS, GATA6, GPR78, HIPK2, HRAS, KAT2B*
hsa-miR-181a-5p	*ABCG2, AHR, ATG5, ATM, BCL2, BCL2L11, C12ORF29, CD4, CDKN1B, CDX2, COL16A1, DDIT4, DDX3X, DUSP5, DUSP6, E2F5, EREG, FOS, GATA6, GPR78, HIPK2, HRAS, IFNG, KAT2B, KLF6, KRAS, MAP2K1, MAPK1, MCL1, MOSPD1, MTMR3, NLK, NOTCH, PBX3, PGR, PHACTR2, PHACTR4, PHLDA1, PLAG1, PPP3CA, PRAP1, PRKCD, PROX1, PTPN11, PTPN22, RALA, RAP1B, RGS5, RNF2, SASH1, SIRT1, SRGAP1, STAT3, TERT, TGFBR1, TGFBRAP1, TIMP1, TWIST1, UBL3, WIF1, XIAP, ZNF763*
hsa-miR-181c-5p	*BCL2, BMPR2, BTBD3, CDX2, DPYSL2, GATA6, IL2, KRAS, MECP2, NLK, NOTCH2, NOTCH4, RAP1B, SIRT1, TRIM2*
hsa-miR-224-5p	*AP2M1, API5, BCL2, CD40, CDC42, CDH1, CXCR4, DIO1, DPYSL2, EDNRA, EYA4, FOSB, HOXD10, KLK10, KRAS, MBD2, NCOA6, NIT1, PAK2, PEBP1, PHLPP1, PTX3, SERPINF2, SMAD4, TCEAL1, TPD52, TRIB1*
hsa-miR-342-3p	*ANKRD49, BMP7, C20orf11, CAMK2N1, CPEB4, DNMT1, GEMIN4, ID4, RMND5A, SREBF1, SREBF2, SYNPO2L, TIAM1*
hsa-miR-452-5p	*BMI1, CDKN1B, DPYSL2, KRAS, LEF1, MMP2, TCF4, THRB, TM7SF4*

Supplementary Table S3: Primers used in this study.

See Supplementary File 2

Supplementary Table S4: Antibodies used in this study

Anti-protein	Host	Dilution and application	Supplier
OCT4	Rabbit	1:100, Immunostaining	Abcam
SOX2	Rabbit	1:1000, Immunostaining	Abcam
SSEA4	Mouse	1:100, Immunostaining	Abcam
TRA-1-60	Mouse	1:500, Immunostaining	Abcam
GAPDH	Rabbit	1:5000, Immunoblotting	Bioworld
MERTK	Rabbit	1:1000, Immunoblotting	Cell Signaling Technology
LRAT	Rabbit	1:1000, Immunoblotting	Abcam
ZO-1	Rabbit	1:200, Immunoblotting	Invitrogen
Keratin 18	Rabbit	1:10000, Immunoblotting	Abcam
RLBP1	Rabbit	1:1000, Immunoblotting	Abcam
β-Catenin	Rabbit	1:1000, Immunoblotting	Cell Signaling Technology
p-AKT2$^{(Ser474)}$	Rabbit	1:1000, Immunoblotting	Cell Signaling Technology
AKT2	Rabbit	1:1000, Immunoblotting	Cell Signaling Technology
p-mTOR$^{(Ser2448)}$	Rabbit	1:1000, Immunoblotting	Cell Signaling Technology
mTOR	Rabbit	1:1000, Immunoblotting	Cell Signaling Technology
p-p70S6K$^{(Thr389)}$	Mouse	1:1000, Immunoblotting	Cell Signaling Technology
p70S6K	Rabbit	1:1000, Immunoblotting	Cell Signaling Technology
RPE65	Mouse	1:250, Immunostaining	Millipore

Supplementary Table S5: Sequences of mimics and inhibitors used in this study

Mimic/Inhibitor	Sequence (5’→3’)
NC-mimic	UUCUCCGAACGUGUCACGUTT
NC-inhibitor	CAGUACUUUUGUGUAGUACAA
hsa-miR-184 mimic	UGGACGGAGAACUGAUAAGGGU
hsa-miR-184 inhibitor	ACCCUUAUCAGUUCUCCGUCCA
dre-miR-184 mimic	UGGACGGAGAACUGAUAAGGGC
dre-miR-184 inhibitor	GCCCUUAUCAGUUCUCCGUCCA
hsa-miR-302d mimic	UAAGUGCUUCCAUGUUUUGGUGA
hsa-miR-302d inhibitor	ACUUUAACAUGGAGGCACUUGC
AKT2-siRNA	CCATGAATGACTTCGACTA
