# Supplementary material for: MicroRNA-184 promotes differentiation of the retinal pigment epithelium by targeting the AKT2/mTOR signaling pathway
Source: Oncotarget. 2016 Jul 13;7(32):52340–53. doi: 10.18632/oncotarget.10566 (PMC5239556; doi:10.18632/oncotarget.10566)
Supplement: Supplementary file 2 [file oncotarget-07-52340-s002.docx]

| **Table S1. A collection of differentially expressed miRNAs** | | | | |
| --- | --- | --- | --- | --- |
| **miRNA** | **Expression Change** | **Mean relative expression of hiPSC-RPE to hiPSC** | | |
|  |  | **30 dpd** | **60 dpd** | **90 dpd** |
| hsa-miR-184 | Up-regulated | 10.7789 | 67.8424 | 149.1824 |
| hsa-miR-449a | Up-regulated | 7.6388 | 18.8387 | 64.0695 |
| hsa-miR-449b-5p | Up-regulated | 6.884 | 15.2824 | 48.6728 |
| hsa-miR-224-3p | Up-regulated | 5.4761 | 9.006 | 31.3521 |
| hsa-let-7e-3p | Up-regulated | 4.2898 | 6.738 | 7.2729 |
| hsa-miR-100-5p | Up-regulated | 2.294 | 7.2687 | 9.9977 |
| hsa-miR-125b-2-3p | Up-regulated | 5.1087 | 22.9935 | 36.2407 |
| hsa-miR-181a-3p | Up-regulated | 4.7607 | 13.7264 | 15.8195 |
| hsa-miR-181a-5p | Up-regulated | 9.0335 | 11.069 | 11.8199 |
| hsa-miR-181c-3p | Up-regulated | 3.7837 | 13.1886 | 16.1596 |
| hsa-miR-181c-5p | Up-regulated | 15.4346 | 18.9497 | 22.2809 |
| hsa-miR-224-5p | Up-regulated | 11.1352 | 15.3199 | 28.5062 |
| hsa-miR-342-3p | Up-regulated | 2.1086 | 3.6532 | 4.4656 |
| hsa-miR-452-5p | Up-regulated | 4.8348 | 14.4024 | 15.6914 |
| hsa-miR-302d-3p | Down-regulated | 0.0796 | 0.0011 | 0.0003 |
| hsa-miR-1273f | Down-regulated | 0.4474 | 0.3564 | 0.1583 |
| hsa-miR-20b-5p | Down-regulated | 0.4803 | 0.3437 | 0.1431 |
| hsa-miR-4430 | Down-regulated | 0.4772 | 0.3334 | 0.1576 |
| hsa-miR-20b-3p | Down-regulated | 0.3419 | 0.1923 | 0.0458 |
| hsa-miR-1244 | Down-regulated | 0.1891 | 0.1861 | 0.0428 |
| hsa-miR-382-5p | Down-regulated | 0.2239 | 0.1498 | 0.0474 |
| hsa-miR-1202 | Down-regulated | 0.4532 | 0.2827 | 0.1821 |
| hsa-miR-122-5p | Down-regulated | 0.0148 | 0.0124 | 0.0088 |
| hsa-miR-127-3p | Down-regulated | 0.0737 | 0.037 | 0.0255 |
| hsa-miR-134-5p | Down-regulated | 0.2768 | 0.1636 | 0.1188 |
| hsa-miR-1908-5p | Down-regulated | 0.4763 | 0.3038 | 0.2327 |
| hsa-miR-302a-5p | Down-regulated | 0.0394 | 0.001 | 0.0009 |
| hsa-miR-302a-3p | Down-regulated | 0.0384 | 0.0006 | 0.0005 |
| hsa-miR-302c-5p | Down-regulated | 0.0372 | 0.0005 | 0.0004 |
| hsa-miR-3175 | Down-regulated | 0.4171 | 0.2004 | 0.1914 |
| hsa-miR-3180-3p | Down-regulated | 0.4639 | 0.3256 | 0.238 |
| hsa-miR-3180 | Down-regulated | 0.4174 | 0.2949 | 0.1818 |
| hsa-miR-3185 | Down-regulated | 0.3178 | 0.1847 | 0.1542 |
| hsa-miR-3187-3p | Down-regulated | 0.2932 | 0.1751 | 0.0882 |
| hsa-miR-3188 | Down-regulated | 0.3045 | 0.1858 | 0.1724 |
| hsa-miR-3196 | Down-regulated | 0.4126 | 0.2687 | 0.229 |
| hsa-miR-3197 | Down-regulated | 0.4766 | 0.3677 | 0.3199 |
| hsa-miR-3178 | Down-regulated | 0.3282 | 0.2228 | 0.1856 |
| hsa-miR-3621 | Down-regulated | 0.4878 | 0.2399 | 0.1748 |
| hsa-miR-3622a-5p | Down-regulated | 0.2538 | 0.1754 | 0.1346 |
| hsa-miR-3648 | Down-regulated | 0.1272 | 0.0792 | 0.0683 |
| hsa-miR-3656 | Down-regulated | 0.3929 | 0.1872 | 0.1829 |
| hsa-miR-373-5p | Down-regulated | 0.2584 | 0.0345 | 0.0202 |
| hsa-miR-379-5p | Down-regulated | 0.1171 | 0.0555 | 0.0357 |
| hsa-miR-3940-5p | Down-regulated | 0.3829 | 0.22 | 0.1809 |
| hsa-miR-409-5p | Down-regulated | 0.2515 | 0.2005 | 0.1815 |
| hsa-miR-433-3p | Down-regulated | 0.153 | 0.0806 | 0.0625 |
| hsa-miR-4417 | Down-regulated | 0.1505 | 0.1152 | 0.0829 |
| hsa-miR-4321 | Down-regulated | 0.3581 | 0.2367 | 0.0984 |
| hsa-miR-4322 | Down-regulated | 0.2705 | 0.2009 | 0.0541 |
| hsa-miR-4327 | Down-regulated | 0.4158 | 0.2983 | 0.2149 |
| hsa-miR-4428 | Down-regulated | 0.4964 | 0.4474 | 0.1752 |
| hsa-miR-4449 | Down-regulated | 0.3028 | 0.2133 | 0.1757 |
| hsa-miR-4486 | Down-regulated | 0.4515 | 0.3357 | 0.2487 |
| hsa-miR-4488 | Down-regulated | 0.4609 | 0.3173 | 0.2601 |
| hsa-miR-4492 | Down-regulated | 0.3044 | 0.1211 | 0.1166 |
| hsa-miR-4498 | Down-regulated | 0.3043 | 0.2768 | 0.1975 |
| hsa-miR-4505 | Down-regulated | 0.299 | 0.1573 | 0.1395 |
| hsa-miR-4508 | Down-regulated | 0.3643 | 0.2132 | 0.1845 |
| hsa-miR-4516 | Down-regulated | 0.3909 | 0.2934 | 0.2825 |
| hsa-miR-4634 | Down-regulated | 0.3242 | 0.2366 | 0.2004 |
| hsa-miR-4647 | Down-regulated | 0.274 | 0.1365 | 0.1204 |
| hsa-miR-4651 | Down-regulated | 0.3746 | 0.201 | 0.1684 |
| hsa-miR-4656 | Down-regulated | 0.4083 | 0.2381 | 0.188 |
| hsa-miR-4665-5p | Down-regulated | 0.3916 | 0.2665 | 0.1727 |
| hsa-miR-4674 | Down-regulated | 0.3597 | 0.2687 | 0.24 |
| hsa-miR-4687-3p | Down-regulated | 0.407 | 0.2688 | 0.2549 |
| hsa-miR-4689 | Down-regulated | 0.386 | 0.215 | 0.2041 |
| hsa-miR-4690-5p | Down-regulated | 0.3786 | 0.2513 | 0.1699 |
| hsa-miR-4721 | Down-regulated | 0.4133 | 0.2809 | 0.242 |
| hsa-miR-4725-3p | Down-regulated | 0.4776 | 0.436 | 0.1365 |
| hsa-miR-4734 | Down-regulated | 0.2849 | 0.1945 | 0.1407 |
| hsa-miR-4735-3p | Down-regulated | 0.475 | 0.2663 | 0.246 |
| hsa-miR-4741 | Down-regulated | 0.3367 | 0.1984 | 0.168 |
| hsa-miR-4745-5p | Down-regulated | 0.4223 | 0.3384 | 0.2323 |
| hsa-miR-4749-5p | Down-regulated | 0.3942 | 0.301 | 0.2383 |
| hsa-miR-4758-5p | Down-regulated | 0.2504 | 0.1443 | 0.1381 |
| hsa-miR-4788 | Down-regulated | 0.4536 | 0.392 | 0.3852 |
