# Supplementary material for: MicroRNA-184 promotes differentiation of the retinal pigment epithelium by targeting the AKT2/mTOR signaling pathway
Source: Oncotarget. 2016 Jul 13;7(32):52340–53. doi: 10.18632/oncotarget.10566 (PMC5239556; doi:10.18632/oncotarget.10566)
Supplement: Supplementary file 3 [file oncotarget-07-52340-s003.docx]

| **Table S3. Primers Used in This Study** | | |
| --- | --- | --- |
| **Gene/Plasmid** | **Forward Primer (5'→3')** | **Reverse Primer (5'→3')** |
| **For Expression Study in Human Cell Line** | | |
| *GAPDH* | CAGCCTCAAGATCATCAGCA | TGTGGTCATGAGTCCTTCCA |
| *NANOG* | ACAGGTGAAGACCTGGTTCC | CTGAGGCCTTCTGCGTCAC |
| *POU5F1* | GCAAAACCCGGAGGAGTC | TGGCTGAATACCTTCCCAAA |
| *SOX2* | CACAACTCGGAGATCAGCAA | GGGCAGCGTGTACTTATCCT |
| *MITF* | AGCGTCCTGTATGCAGATGG | CCGAGACAGGCAACGTATTT |
| *PAX6* | ACCGGTTTCCTCCTTCACAT | GGGTTGCATAGGCAGGTTAT |
| *RPE65* | TACAGAAAGCACTGAGTTGAGC | CCATTTAGTAAGTCCACATTCATTTCC |
| *RLBP1* | GCTGCTCAGAGGCTATGTGA | TGCCTGCAAGATCTCATCAA |
| *MERTK* | AGTGCAGGGATTTCCAAAGA | GGGGCATAATCTACCCAACC |
| *BEST1* | CCTGCTGAACGAGATGAACA | CCACAGTCACCACCTGTGTA |
| *CDH2* | ATGCCAAGCCAGGACAGATA | AATCCCCGCTGTGTTGTTT |
| *TJP1* | CTTCCAGAACCAAAGCCTGT | ATGCTGGGCCGAAGAATC |
| *AKT2* | TAGCAGAATGCCAGCTGATG | ATCCACTCCTCCCTCTCGTC |
| *LRAT* | TCAGACCTACCAGTTCTGCAA | CAAACAGGGTCACCGACTG |
| *KRT18* | GGAGACTTCAAACTCCAGGATG | TCTGTGCTTGAGGACACAAC |
| *CTNNB1* | GGGATCAAATCTGACACCAAA | CCTCAGCTCCCTGGTCAAT |
| **For Expression Study in Zebrafish** | | |
| *gapdh* | GTCTTGAGAAACCTGCCAAG | AGTGGACACAACCTGGTGCT |
| *pax6a* | CAACGCCAGTATCCTTTACG | GGCTGGGTTGCATAGGAAG |
| *rpe65c* | ATCCCGCCACTACAAGAAGA | TGCCAAAGCTGTGAACGTAG |
| *rlbp1b* | TGGACATGTTGCAGGACTCT | ACAAACACCCTTTCCAGCAG |
| *lrat* | TCAGACCTACCAGTTCTGCAA | CAAACAGGGTCACCGACTG |
| *mertka* | GCTACATCGCGGTCAGAGAG | AACGCCCTCAGTGGTGTTAC |
| *best1* | GTGGGCTGGCTTAAGGTTG | CTGACAGCAGAGACACCTGTAAA |
| *krt18* | GGAGACTTCAAACTCCAGGATG | TCTGTGCTTGAGGACACAAC |
| *cdh2* | TCCTGTTAGATATTCTGTTGAATGGA | TGGACAGCTGGGGATTGTTA |
| *tjp1b* | CTGCTGCAGGAGAATGACAG | TTACGCTTTCTCCTGATGGTT |
| *ctnnb1* | GGGATCAAATCTGACACCAAA | CCTCAGCTCCCTGGTCAAT |
| **For Plasmid Construction** | | |
| AKT2^WT^ | CGAGCTCGTGTGTGTGCATGTGTGTTTGTGTGAGG | CCCTCGAGGGATATGAAGACGAGGAGAAAGGCCAGT |
|  | *SacI* | *XhoI* |
| AKT2^MU^ | CGAGCTCGTGTGTGTGCATGTGTGTTTGTGTGAGG | CCCTCGAGGGGGAGAAAGGCCAGTAGCGGCGTAGA |
|  | *SacI* | *XhoI* |
| AcFlag-AKT2 | GGATCCATGAATGAGGTGTCTGTCATCAAAG | TCTAGACTCGCGGATGCTGGCC |
|  | *BamHI* | *XbaI* |
| Bold font indicates the title and subtitles, and italic font indicates the genes. | | |
